# 

**Published:** 1820-08

**Authors:** 


### MONTHLY CATALOGUE OF MEDICAL BOOKS.

A Dissertation on Infanticide, in its Relations to Physiology and Jurisprudence. By Wm. Hutchinson, M.D. F.L.S. 8vo. 5s. 6d.

Principles of Military Surgery; comprising Observations on the Arrangement, Police, and Practice of Hospitals; and on the History, Treatment, and Anomalies, of Variola and Syphilis. Illustrated with Cases, Dissections, and Engravings. By John Hennen, M.D. F.R.S.E. Deputy-Inspector of Military Hospitals. Second Edition, with numerous Additions. 8vo. 18s. boards.

Cases in Surgery, selected from the Records of the Author's Practice at the St. George's and St. James's Dispensary; and illustrating the Nature and Modes of Treatment of Strumous or Scrofulous Ophthalmia; the Sedative Powers of Tartar-Emetic in the Cure of Local Inflammation, when administered internally. By Henry Jeffreys, Esq. Senior Surgeon to the St. George's and St. James's Dispensary. 8vo. 8s. boards.

Symphylakesis, vulgarly called Cancer; being the Substance of a Lecture on that Disease, in a Series of Medical Tuition. By T. Parkinson, M.D. Author of Synopsis Zoo-Nosologiæ. 12mo. 1s. 6d.

Introductory Lecture to a Course of Botanical Lectures, Excursions, and Demonstrations; delivered in Edinburgh. By James Robinson Scott, F.R.S.E. F.L.S. &c. 8vo. 2s.

Medical Transactions, by the College of Physicians in London. Vol. VI. 8vo. 12s. boards.

A History of the Variolous Epidemic which occurred at Norwich in the year 1819, and destroyed 530 Individuals: with an Estimate of the Protection afforded by Vaccination, and a Review of past and present Opinions upon Chicken-pox and modified Small-pox. By John Cross, Member of the Royal College of Surgeons, London, &c. 8vo. 9s. boards.

[HIGHLEY AND SON, FLEET-STREET.]

